# Sensorimotor and body perception assessments of nonspecific chronic low back pain: a cross-sectional study

**DOI:** 10.1186/s12891-021-04269-7

**Published:** 2021-04-26

**Authors:** R. Meier, C. Emch, C. Gross-Wolf, F. Pfeiffer, A. Meichtry, A. Schmid, H. Luomajoki

**Affiliations:** 1 Prodorso, Walchestrasse 15, CH-8006 Zurich, Switzerland; 2 Zurich University of Applied Sciences (ZHAW), School of Health Professions, Institute of Physiotherapy, Katharina-Sulzer-Platz 9, CH-8400 Winterthur, Switzerland; 3 Physiotherapie im Schutzengel AG, Allmendstrasse 1, CH-6300 Zug, Switzerland; 4 Physiotherapie im Sonnenheim, Sonnenheim 8, CH-6344 Meierskappel, Switzerland; 5 Nuffield Department of Clinical Neurosciences, University of Oxford, Oxford, OX3 9DU UK

**Keywords:** Chronic nonspecific low back pain, Back-photo assessment, Two-point discrimination, Movement control test

## Abstract

**Background:**

Low back pain (LBP) is one of the most common musculoskeletal disorders, causing significant personal and social burden. Current research is focused on the processes of the central nervous system (particularly the sensorimotor system) and body perception, with a view to developing new and more efficient ways to treat chronic low back pain (CLBP). Several clinical tests have been suggested that might have the ability to detect alterations in the sensorimotor system. These include back-photo assessment (BPA), two-point discrimination (TPD), and the movement control tests (MCT).

The aim of this study was to determine whether the simple clinical tests of BPA, TPD or MCT are able to discriminate between nonspecific CLBP subjects with altered body perception and healthy controls.

**Methods:**

A cross-sectional study was conducted. At one point in time, 30 subjects with CLBP and 30 healthy controls were investigated through using BPA, TPD and MCT on the lower back. Correlations among the main covariates and odds ratios for group differences were calculated.

**Results:**

MCT showed an odds ratio for the presence of CLBP of 1.92, with a statistically significant *p*-value (0.049) and 95%CI. The TPD and BPA tests were unable to determine significant differences between the groups.

**Conclusions:**

Of the three tests investigated, MCT was found to be the only suitable assessment to discriminate between nonspecific CLBP subjects and healthy controls. The MCT can be recommended as a simple clinical tool to detect alterations in the sensorimotor system of nonspecific CLBP subjects. This could facilitate the development of tailored management strategies for this challenging LBP subgroup. However, further research is necessary to elucidate the potential of all the tests to detect alterations in the sensorimotor system of CLBP subjects.

**Trial registration:**

No trial registration was needed as the study contains no intervention. The study was approved by the Swiss Ethics Commission of Northwest and Central Switzerland (EKNZ) reference number 2015–243.

## Background

Low Back Pain (LBP) is one of the most common musculoskeletal disorders and causes significant personal and social burden [1]. Currently, 85% of LBP cases are classified as nonspecific LBP, meaning that there are no specific structural causes that can solely explain the symptoms [2]. Due to the complexity of the situation, outcomes for unimodal treatments are poor [1]. Thus, good strategies to manage nonspecific LBP, particularly when a chronic pain state exists, include conservative and invasive treatments. Nevertheless, because of the nature of the nonspecific structural causes, a tailored management strategy for nonspecific chronic LBP treatment remains a major challenge [3–5]. Chronic pain is defined as pain lasting longer than 3 months [6].

Recent neuroimaging studies have demonstrated neurochemical, structural and functional alterations in the primary sensory cortex in subjects with chronic low back pain (CLBP) [2, 4, 5]. These findings support the emerging evidence that the central nervous system (CNS) processes also contribute to CLBP [7, 8]. CLBP may increase sensitivity in the spinal cord and the cortex, leading to the amplification of peripheral inputs. Furthermore, the inhibitory mechanisms of the CNS demonstrate reduced functionality in CLBP subjects [5, 7, 9, 10]. These factors may contribute to the sensorimotor changes and altered body perceptions, as well as to reduced grey matter volume in the somatosensory cortex [5, 7, 9, 10]. Identifying such alterations through neuroimaging assessments are costly and difficult to access. Therefore, simple clinical tests to identify such alterations in the sensorimotor system are increasingly important to physicians and physical therapists to facilitate the development of tailored management strategies for patients in a chronic low back pain state, especially for the challenging subgroup of the nonspecific CLBP group. Further research on relevant simple clinical tools is needed.

Several clinical assessments are thought to be capable of detecting changes in the sensorimotor system and body perception. It was decided to investigate three of these common assessment tools in this paper due to their practicability in clinical work. The aim of this replication study was to determine whether the simple clinical tests are able to discriminate between nonspecific CLBP subjects with altered body perception and healthy controls. Therefore, two-point discrimination (TPD), movement control tests (MCT), as well as the more recent back-photo assessment (BPA) were included.

The first test BPA, which is a visual approach capable of testing body perception and perceived body image [8]. The alternative visual approach of body image drawing was dismissed because of its poor performance in a previous study [11]. BPA uses photographs to reflect a person’s lower back at modified widths. The subject is then required to identify the original, unmodified photograph of their back from the various versions. This method has been used previously only for limbs in patients with complex regional pain syndrome (CRPS) and is thought to show changes in the primary sensory cortex S1 representation [8]. However, BPA has not been validated for CLBP patients. Recent evidence has demonstrated altered body image perception in subjects with CLBP when assessed by completing a partial drawing of their back silhouette with the body image drawing approach. However, the CLBP subjects were unable to clearly outline their trunk in the painful area [12].

The second test investigates TPD on the lower back, examining the tactile acuity of LBP patients. Subjects with LBP have been shown to demonstrate increased TPD values compared to healthy controls [13, 14]. TPD has been proposed as a surrogate measure for changes in the somatosensory cortex (S1) [9, 15–17].

The third test addresses the reduced perception of the spine [18] displayed by CLBP patients, which affects movements controlled by the central nervous system [10, 19]. MCT are common in identifying possible deficits in motor control [20, 21] and can discriminate between LBP subjects and healthy controls [10, 18–21]. Additionally, movement control impairment (MCI) and TPD outcomes appear to be associated [17].

The aim of this study was to determine whether BPA, TPD and MCT, which are thought to reflect sensorimotor changes, were able to discriminate between subjects with nonspecific CLBP and altered body perception and healthy controls. This would enable physicians to detect alterations in the sensorimotor system of nonspecific CLBP subjects using simple clinical tools and, thus, facilitate the development of tailored management strategies for this challenging LBP subgroup.

## Methods

### Design

A cross-sectional study was conducted. The study was approved by the Swiss Ethics Commission of Northwest and Central Switzerland (EKNZ) (reference number 2015–243). All participants gave their informed written consent prior to study start and all procedures conformed to the Declaration of Helsinki.

### Participants

A convenience sample of 60 participants, 30 subjects with nonspecific CLBP and 30 healthy controls, were recruited from three outpatient physiotherapy clinics in Central Switzerland. The private clinics were selected according to the quantity of patients with relevant cases. The CLBP and control groups were matched for gender and age, but no further subgrouping was made. Inclusion criteria were: 1) age over 18 years; 2) proficient in the written and spoken German language; 3) no current pregnancy, or pregnancy in the past 6 months; 4) at least four points on the Roland Morris Disability Questionnaire (RMDQ) - indicating at least moderate disability due to LBP; and 5) the presence of CLBP - defined as at least 3 months of unilateral or bilateral nonspecific LBP. Exclusion criteria were: 1) clinical bedside signs of nerve root pain, or evidence of specific spinal pathology (e.g., malignancy, fracture, infection, inflammatory joint or bone disease; and 2) surgery on the lower back in the past 6 months. Healthy controls were excluded when they had any history of LBP in the past 6 months, or a period of LBP of more than 1 month in the past.

### Questionnaires

Basic demographic data, − gender, age, weight, height, body mass Index (BMI), affected side, pain duration and pain intensity - were obtained from all participants. The Roland Morris Disability Questionnaire (RMDQ) [22, 23] was used to screen for eligibility, with a score of at least 4 points on this scale being necessary for inclusion. Subsequently, participants completed the Fear Avoidance Belief Questionnaire (FABQ) [24], Fremantle Back Awareness Questionnaire (FreBaQ) [23] and Hospital Anxiety and Depression Scale (HADS) [25–27]. These questionnaires had been previously validated in the German language.

Subjects of the CLBP group were asked additionally to report the locality of their pain (bilateral, left-sided, or right-sided), its duration and mean intensity, using a numeric rating scale (NRS). This scale ranges from 0 (no pain) to 100 (worst pain) [28].

### Assessments

Examiners, blinded to the participants’ condition, recorded the results of the BPA, TPD and MCT. The examiner who produced all the back photos made no assessments and was blinded to the subjects’ group. The two examiners undertaking the physical testing were also blinded to the subject grouping. The latter were both experienced physiotherapists with a musculoskeletal physiotherapy specialisation. However, the tests were not explicitly trained.

### Back-photo assessment

**Fig. 1:**
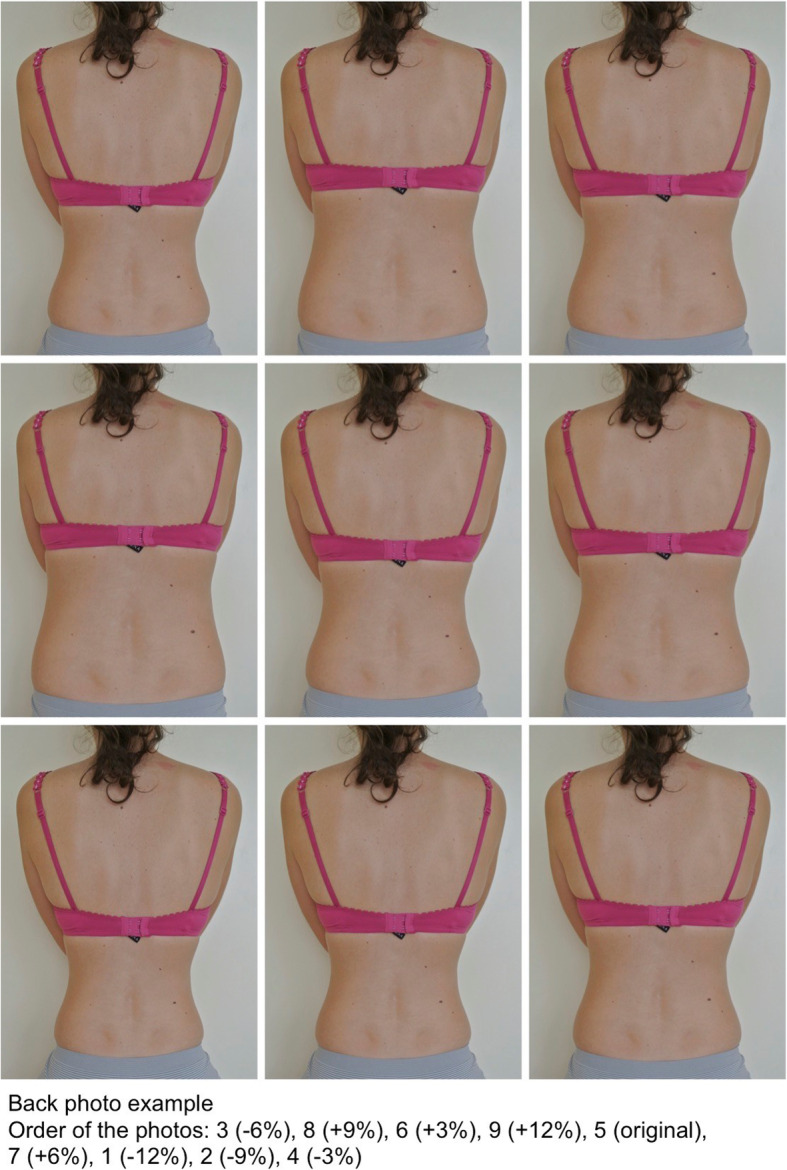
Back-photo assessment (BPA)

BPA had previously been used to assess limbs in a population with complex regional pain syndrome [8]. The lower back was the focus of this study. The lumbar vertebra four was marked on the skin of the subject and a photograph was taken from the middle part of the gluteal area to the occipital part of the skull to depict the participant’s back in a standing position. The photograph was then modified at level L4 in steps of 3% enlargement and shrinkage, using the GNU Image Manipulation Program (GIMP 2.8.14 for OS X). We chose to use 9 different sizes, instead of 7 used by an earlier study [8] and therefore increased the sizes only by 3% instead of 5%. The maximal limit was set at ±12%. This resulted in eight modified photos, plus the original photo for each participant. The photos were allocated numbers from 1 to 9 in order of the extent of modification. Numbers 1 to 4 were allocated to the shrunken photos, with 1 representing the photo with the maximal shrinkage of − 12% (Fig. 1). Number 5 was given to the original, unmodified photo. Numbers 6 to 9 were allocated to the enlarged photos, with 9 representing the photo with the maximal enlargement of + 12%. The photos were arranged on a sheet of paper in a randomised sequence, with the same sequence being used for all participants. The participants were then requested by the examiner to identify the original photo of their back. The BPA outcome value was in the range from 0 to 4, reflecting the number of steps of modification between the original photo and the photo selected by the subject, irrespective of direction.

### Two-point discrimination

**Fig. 2:**
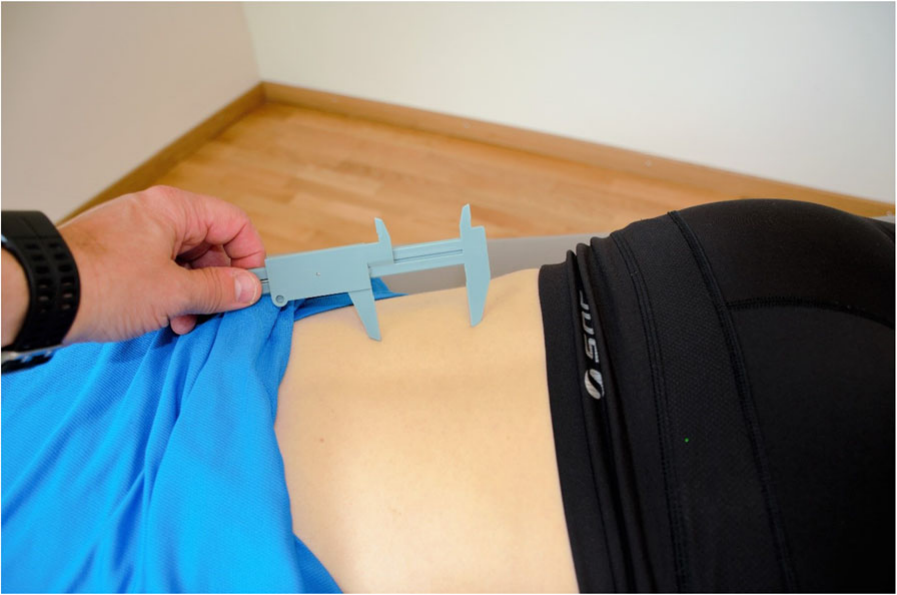
Two point discrimination test (TPD)

TPD is a reliable intra-rater measure to detect altered tactile acuity [29]. TPD measurements were taken using a plastic calliper, according to an established protocol [15, 17, 30] (Fig. 2). The participant lay prone and unable to see the calliper. An examiner measured both the horizontal and vertical TPD bilaterally on the participant’s lower back at level L4 [29]. The calliper tip distance ranged from 100 mm to 5 mm, with the test started at the maximum spread. For every correct detection, the spread distance was decreased by 10 mm. Conversely, for every incorrect detection, the spread distance was increased by 5 mm. This procedure was repeated three times in descending and ascending order and the average of the smallest distance between the calliper tips at which the participant was still able to discriminate between the two separate points was recorded as the TPD value [17].

### Movement control test

**Fig. 3:**
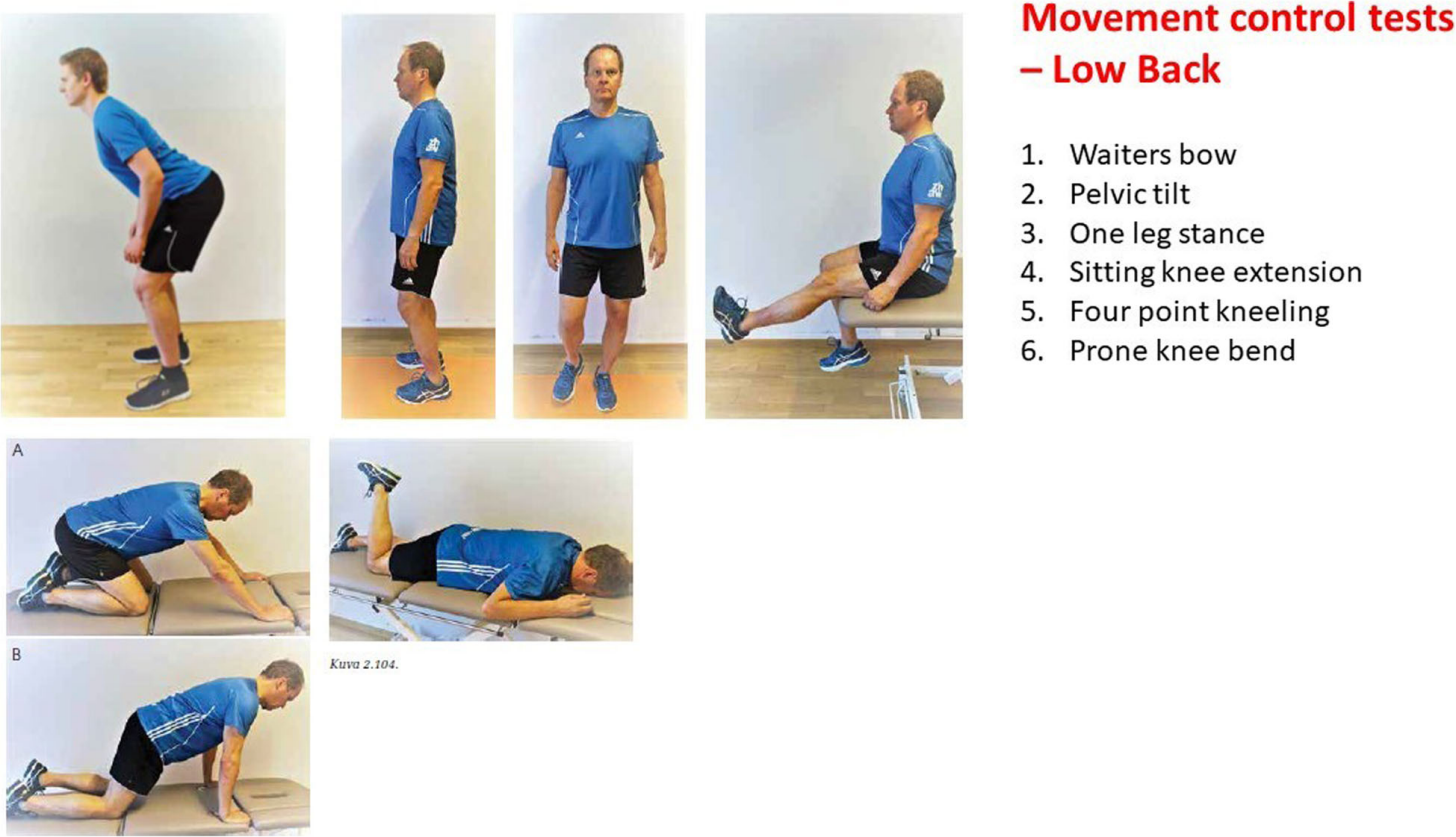
Movement control test batterie of the low back

MCI of the lumbar spine was evaluated using a battery of six tests designed to ascertain the movement control of the back (Fig. 3). The MCT battery has been shown to be a reliable tool in detecting impaired lumbopelvic control [20, 21, 31]. Before the assessment start, the examiner explained the six specific movement tasks of the MCT to the subject. The following protocol was used for each specific movement. At the beginning, the subject was verbally instructed by the examiner to perform a specific movement. A correctly performed movement was rated as a negative outcome for this task and the examiner continued directly to the next specific movement. When the specific movement was not performed correctly, the examiner provided verbal corrections. If the movement was still performed incorrectly, then the examiner demonstrated the correct movement. When, with this support, the subject was able to perform the specific movement correctly, then the outcome for this task was rated negative and the examiner went on to verbally instruct the next specific movement. When the subject was unable to perform the specific movement correctly, even with support, then the outcome for this task was rated positive and the examiner went on to verbally instruct the next specific movement. The tasks outcomes were recorded and a final score calculated ranging from 0 (all movements performed correctly) to 6 (no movement performed correctly). The subjects’ scores were noted as the outcome values for MCT. A detailed description of the MCT test battery and the definition of ratings can be found elsewhere [20].

### Statistical analysis

All statistical analyses were performed using R (version 3.2.3) [32]. Normality of data was determined by inspection of histograms. Demographics, questionnaire data, BPA, TPD and MCT were analysed with descriptive statistics. Spearman correlations were used for independent variables (BPA, TPD, MCT, FreBAQ, FABQ, HADS, Age, Body height, Body weight, BMI). Multiple logistic regressions were applied with conditional likelihood to determine associations between the main outcomes (BPA, TPD and MCT) with the presence of CLBP. Concerning the goodness-of-fit test criteria for applying a multiple logistic regression, it was not applicable to run a Hosmer-Lemesbow test, since this is only suited to unconditional logistic regression. The Hosmer-Lemesbow test is computed with the expected probabilities of an event. These are unknown for conditional logistic regression because the model omits the unobserved intercept for each individual. As a compromise, a likelihood-ratio test was added, as well as concordance statistics. The concordance is defined as the probability that the prediction goes in the same direction as the actual data. CI > 0.5 implies a good predictive ability. Checks for outliers/influential data and for collinearity were performed. There was no evidence of outliers/influential data and there was low collinearity. To check for multicollinearity, variance inflation factors (VIf) were computed, with cut-off value 5 [33]. The log odds of the presence of CLBP were modelled with six covariates. These covariates were BPA, TPD (left and right horizontal TPD, left and right vertical TPD) and MCT. The objective was to quantify the effect of each covariate on the outcome CLBP, reported in odds ratios. The questionnaires revealed no associations with the main outcomes and were not investigated further.

## Results

Not all data were normally distributed. The conducted analysis produced the following values for the data set. Likelihood-ratio test = 6.07 on 6 df, *p* = 0.4, Concordance = 0.7 (se = 0.118). All VIf were smaller than 4.1, thus no VIf was more extreme than the cut-off 5.

**Fig. 4:**
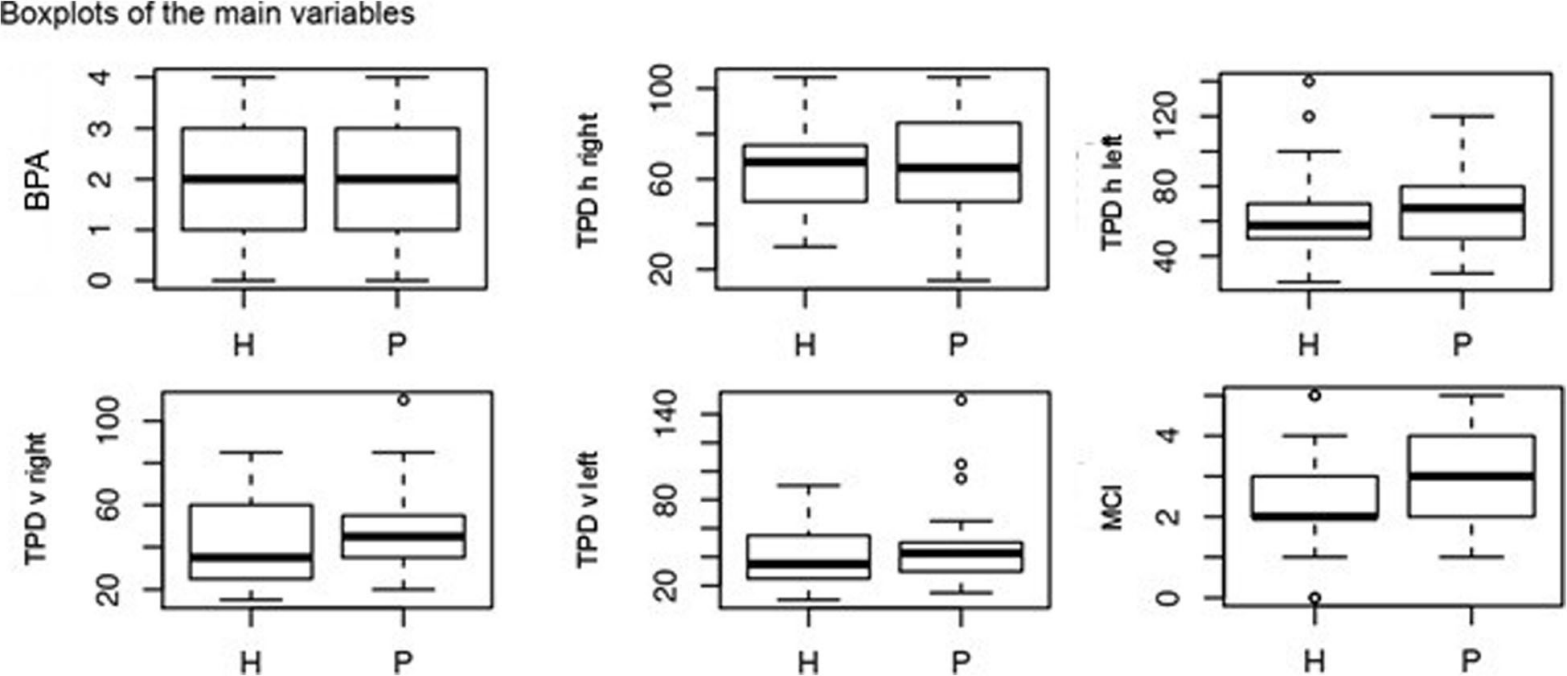
Box plots of the main variables

**Fig. 5:**
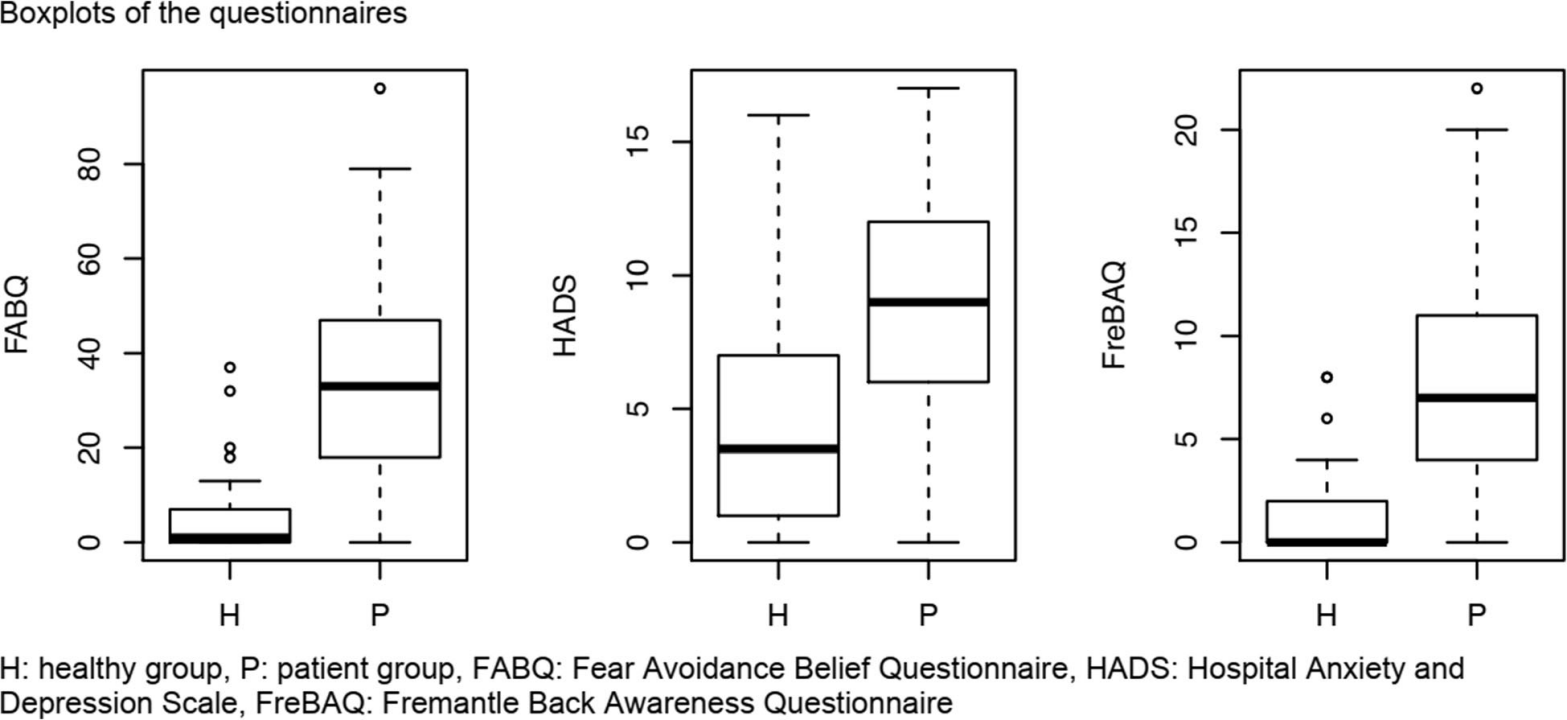
Box plots of the questionnaires

The demographics of both groups were similar at baseline, apart from weight and Body Mass Index (BMI), which were higher in the CLBP group (Table 1). Table 2 contains the results of the assessments. BPA and TPD values were similar for both groups. Table 3 summarises the results of the multiple logistic regression analysis. Significant between-group differences could be demonstrated for MCT only, with an odds ratio of 1.92 for the presence of CLBP and a statistically significant 95%CI of 1.00–3.68. This means that for each point greater on the MCT battery the odds of being a subject with LBP increases 1.92 times. The same result was not found for TPD or BPA. No statistically significant correlations between the independent variables were identified (Table 4). However, the results of the FABQ and FreBaQ demonstrated large between-group differences (Table 2). Figures 4 and 5 display box plots of the main variables and questionnaires.

**Table 1:** Demographic characteristics

Variables	**CLBP group** (*n* = 30)			**Healthy control group** (*n* = 30)	
Affected side (bilateral / left / right)	17 / 3 / 10				
Gender female / male	15/15			15/15	
Variables	*p*-values	Mean	Range	Mean	Range
Age (years)	0.40	52.9 (SD 18.0)	25.0–83.0	51.8 (SD 16.5)	22.0–79.0
Weight (kg)	0.01	81.3 (SD 18.0)	50.0–122.0	72.0 (SD 11.9)	52.0–100.0
Body height (cm)	0.38	172.4 (SD 9.7)	157.0–194.0	173.1 (SD 8.7)	155.0–186.0
Body Mass Index	< 0.02	27.4 (SD 5.8)	16.9–44.6	24.0 (SD 3.0)	18.3–30.2
Pain duration (months)		131.8 (SD 160)	3.0–660.0		
Pain intensity (0–100)		33.4 (SD 20)	4.0–75.0		
RMDQ (0–24)		8.2 (SD 4.1)	4.0–21.0	0.07 (SD 0.3)	0.0–1.0

*RMDQ* Roland Morris Disability Questionnaire

**Table 2:** Outcomes

Variables	**CLBP group** (*n* = 30)		**Healthy control group** (*n* = 30)	
BPA wider	18 (60%)		16 (53%)	
BPA narrower	8 (26.5%)		10 (33.5%)	
BPA original	4 (13.5%)		4 (13.5%)	
Variables	Median	Range	Median	Range
BPA deviation steps (0–4)	2.0 (IQR 2.0)	0.0–4.0	2.0 (IQR 2.0)	0.0–4.0
TPD horizontal right (mm)	65.0 (IQR 33.8)	15.0–105.0	67.5 (IQR 23.8)	30.0–105.0
TPD horizontal left (mm)	67.5 (IQR 30.0)	30.0–120.0	57.5 (IQR 18.8)	25.0–140.0
TPD vertical right (mm)	45.0 (IQR 20.0)	20.0–110.0	35.0 (IQR 33.8)	15.0–85.0
TPD vertical left (mm)	42.5 (IQR 20.0)	15.0–150.0	35.0 (IQR 28.8)	10.0–90.0
MCT positive (0–6)	3.0 (IQR 2.0)	1.0–5.0	2.0 (IQR 1.0)	0.0–5.0
FABQ (0–96)	33 (IQR 28.8)	0–96	1 (IQR 6.8)	0–37
HADS (0–42)	9 (IQR 5.5)	0–17	3.5 (IQR 5.8)	0–16
FreBaQ (0–36)	7 (IQR 6.8)	0–22	0 (IQR 2.0)	0–8

*BPA* Back-Photo Assessment, *TPD* Two-Point Discrimination, *MCT* Movement Control Tests, *FABQ* Fear Avoidance Belief Questionnaire, *HADS* Hospital Anxiety and Depression Scale, *FreBaQ* Fremantle Back Awareness Questionnaire

**Table 3:** Multiple logistic regression

Variables	log odds ratio	Odds ratio	se	z-value	*P*-value	95% CI
BPA	−0.30	0.74	0.29	−1.03	0.30	0.42–1.31
TPD horizontal right	0.01	1.01	0.02	0.40	0.69	0.96–1.06
TPD horizontal left	−0.02	0.98	0.03	−0.56	0.57	0.93–1.04
TPD vertical right	0.03	1.03	0.03	0.92	0.36	0.97–1.10
TPD vertical left	0.01	1.00	0.02	0.08	0.94	0.96–1.05
MCT	0.65	1.92	0.33	1.96	0.05	1.00–3.68

*BPA* Back-Photo Assessment, *TPD* Two-Point Discrimination, *MCT* Movement Control Tests

**Table 4:** Spearman correlations

Variables	TPD h right	TPD h left	TPD v right	TPD v left	MCT	FreBAQ	FABQ	HADS	Age	Body height	Body weight	BMI	BPA
TPD h right	–												
TPD h left	0.79	–											
TPD v right	0.41	0.59	–										
TPD v left	0.32	0.58	0.85	–									
MCT	−0.14	−0.09	−0.09	0.01	–								
FreBAQ	0.02	0.09	0.23	0.17	0.22	–							
FABQ	−0.03	0.08	0.17	0.17	0.23	0.71	–						
HADS	0.08	0.11	0.20	0.02	0.06	0.58	0.59	–					
Age	0.30	0.38	0.21	0.05	−0.21	−0.16	−0.13	0.20	–				
Body height	0.05	0.03	0.04	0.05	0.22	−0.21	−0.12	−0.08	− 0.18	–			
Body weight	0.46	0.42	0.12	0.11	0.25	0.10	0.04	0.20	0.17	0.46	–		
BMI	0.45	0.44	0.18	0.15	0.14	0.22	0.12	0.27	0.28	−0.03	0.85	–	
BPA	0.23	0.26	0.17	0.25	0.09	−0.09	−0.08	0.07	0.15	0.01	0.29	0.35	–

*BPA* Back-Photo Assessment, *TPD* Two-Point Discrimination horizontal vertical, *MCT* Movement Control Tests, *FABQ* Fear Avoidance Belief Questionnaire, *HADS* Hospital Anxiety and Depression Scale, *FreBaQ* Fremantle Back Awareness Questionnaire, *BMI* Body Mass Index, *h* Horizontal, *v* Vertical

## Discussion

### Main findings

The main objective of this study was to examine the ability of three commonly performed clinical tests to discriminate between nonspecific CLBP subjects and healthy controls.

Our results revealed discriminative ability for the MCT, but not for the BPA and TPD tests. Consequently, only the MCT, due to its odds ratio of 1.92 and statistically significant *p*-value (0.049) and 95%CI, can be recommended as a test for the detection of the likelihood of the presence of LBP. However, the 95%CI lower limit was near 1 (1.0002–3.677) and close to not being significant. It cannot be said whether this difference is clinically meaningful.

### Comparison to earlier studies

This finding for MCT confirms previous results, in which the ability was found to discriminate between LBP subjects and healthy controls [10, 18–21]. Luomajoki et al. cited a mean MCT score of 2.21 (out of 6) for subjects with LBP and 0.75 for healthy controls [21]. In contrast, both of the groups in this study showed higher MCT scores: 3.0 for CLBP subjects and 2.0 for healthy controls. An explanation could be that this current study included subjects with CLBP only, whereas Luomajoki et al. also investigated subjects with acute and subacute LBP [21].

Our results for the BPA test differ from the previous research by Moseley et al. (2005), in which a similar approach with CRPS patients was used. Results from this study demonstrated that the subjects in the chronic pain group selected photos with a 7% enlargement of the original photo size [8], compared to our study in which both CLBP subjects and healthy controls tended to choose enlarged photos of their backs, but with no meaningful between-group difference. The BPA results in our study indicate no significant association between an increased BPA score and a higher chance of suffering from CLBP. The divergent results might be partly explained by the different enlargement steps used in the Moseley et al. study. Our photos were modified in 3% steps, whereas Moseley et al. used 5% steps. Also in contrast to our study, the latter demonstrated a correlation between the chosen picture and the duration of symptoms [8]. BPA is a rather novel test for the detection of altered body perception, based on preliminary data from CRPS patients, but which has not yet been validated for CLBP subjects. The focus of this study was the trunk, whereas Moseley et al. examined limbs. Moseley et al. modified photos of the affected hand, thereby allowing a comparison of the person’s two hands. In contrast, modified photos of one area of the back at level L4 were shown in the current study. Hence, the results of the two studies cannot be compared directly. Additional research is needed to improve BPA testing. We recommend that the enlargement steps should be 5% as used by Moseley et al., and the impact of smaller increments on significance be investigated.

Our findings for the TPD test also diverge from those of previous studies. The earlier studies demonstrated the ability of TPD to discriminate between CLBP subjects and healthy controls [15, 17, 34]. Luomajoki and Moseley also observed a correlation between TPD and MCT [17]. In our study, no statistically significant correlation between the TPD and MCT was identified. Additionally, no statistically significant correlations among the independent variables were found. This accords with previous research. Ehrenbrusthoff et al. doubted the similarity of the underlying construct of the TPD and the FreBaQ questionnaire and, thus, questioned the correlation between them [35]. However, recent studies have demonstrated correlations between TPD and body image drawings, a different visual approach with similarities to BPA [12, 36].

### Strengths and limitations

The strength of this study is that it replicates previous studies using MCT and TPD, but also includes the more recent BPA assessment. The latter has not been investigated in this population. It addresses the subject of altered body perception in a chronic pain state when no specific structural causes in the back can solely explain the symptoms. This is a field in which further research is needed.

Our study has some limitations. The investigation of a specific subgroup of patients with LBP, such as those with movement control impairment, might have been more beneficial. Another possible limitation is that the nonspecific CLBP cohort in this study showed a low pain intensity of 33.4/100 NRS and disability of 8.2/24 at baseline. These levels may not have been high enough to result in significant alterations in the sensorimotor system, which could explain the inability of the included tests to demonstrate significant discriminative capability. Nevertheless, it remains unclear as to whether higher pain intensities and disability levels in a nonspecific CLBP cohort, through the detection of potentially larger alterations in the sensorimotor system, would have resulted in greater discriminative ability of the tests. Previous research has shown a change in proprioception due to exercise and, therefore, in movement control [1, 37, 38]. Therefore, whether the activity level CLBP subjects has an impact on outcomes needs to be investigated. It is unclear whether CLBP subjects with higher activity levels would have different outcomes to those with lower activity levels.

A methodological limitation of this study is that two different examiners performed the TPD assessments to maintain assessor blinding. Catley et al. (2013) questioned the inter-rater reliability of TPD on the lower back [29]. The raters in this study did not explicitly train the methodology of the measurements with each other and this could have influenced the results.

Neither selection bias can be negated as the patients were by sample of convenience and from only three practises. It might have been also wise to subgroup patients according to system introduced by O’Sullivan [2].

It is also unclear whether the difference in MCT outcomes between the study groups should be viewed as clinically important, since the levels of minimal clinically meaningful differences have yet to be reported. The raters also did not explicitly train with each other for the MCT prior to the testing. However, both raters were educated to at least master level in musculoskeletal physiotherapy and were experienced in MCT testing.

## Conclusion

The ability of BPA, TPD and MCT to discriminate altered body perception in nonspecific CLBP subjects was investigated. A strength of this study is its focus on altered body perception of the back in a chronic pain state, where no specific structural causes could solely explain the symptoms. This is a field in which further research is necessary.

Only the MCT was shown to be able to in discriminate between nonspecific CLBP subjects (with low to moderate pain and disability levels) and healthy controls. Therefore, MCT can be recommended as a simple clinical tool to detect alterations in the sensorimotor system of nonspecific CLBP subjects and, hence, to facilitate the development of tailored management strategies for this challenging LBP subgroup. However, further research is required to elucidate the potential of other simple clinical tests, such as BPA and TPD, to detect alterations in the sensorimotor system in CLBP subjects.

## Data Availability

The datasets used and/or analysed during the current study are available from the corresponding author on reasonable request.

## Abbreviations

BPA: Back-photo assessment
BMI: Body mass index
CLBP: Chronic low back pain
CRPS: Complex regional pain syndrome
FABQ: Fear Avoidance Belief Questionnaire
FrBaQ: Fremantle Back Awareness Questionnaire
HADS: Hospital Anxiety and Depression Scale
LBP: Low back pain
MCI: Movement control impairment
MCT: Movement control tests
RMDQ: Roland Morris Disability Questionnaire
EKNZ: Swiss Ethics Commission Northwest and Central Switzerland
TPD: Two-point discrimination
NRS: Visual analogue scale
